# What does a pandemic proof health system look like?

**DOI:** 10.1080/16549716.2021.1927315

**Published:** 2021-07-11

**Authors:** Janet Michel, Thomas C Sauter, Marcel Tanner

**Affiliations:** a Insel University Hospital, Department of Emergency Medicine, University of Bern, Bern, Switzerland; b Epidemiology and Public Health Department, Swiss Tropical and Public Health Institute, Basel, Switzerland; c University of Basel, Basel, Switzerland

**Keywords:** Health care system, pandemic proof, COVID-19, societal issues, science vs social media

## Abstract

Switzerland is currently in a lockdown and other lockdowns are looming world-wide. Many countries in the West are now experiencing a third COVID-19 wave. While some scientists are aiming for Zero Covid, calls to learn to live with the virus are becoming prominent as anti-lockdown protests spread across Europe. A health system is defined as all organizations, institutions and resources that produce actions whose primary purpose is to improve health. A health care system on the other hand, is defined as institutions, people and resources involved in delivering health care to individuals. Many countries that have health systems previously thought to be world class, have also been tested, pushed to the edge and in some ways found wanting. The pandemic took all countries by some surprise and the discussion on appropriate national and global strategies is very diverse. Lessons from similar earlier outbreaks seem to suggest, that living and learning to live with the virus could be the way forward. Others argue that the virus is new, not like any other we have fought before, calling for novel ways of containing the virus. Irrespective of standpoint, being a high-, middle- or low-income country, pandemic fatigue is setting in, while new variants are being discovered. It is urgent and unprecedented. The pandemic is here and more pandemics are expected to follow. What does, ‘living with the virus,’ mean in practical terms? The purpose of this viewpoint is to stimulate debate on how we can move towards pandemic proof health care systems.

## Background

Switzerland is currently in a lockdown and other lockdowns are looming world-wide [1–3]. Many countries in the West are now experiencing a third COVID-19 wave. While some scientists are aiming for Zero Covid, calls to learn to live with the virus are becoming prominent as anti-lockdown protests spread across Europe. Zero Covid is defined as reducing infections to a point where it is possible to test, trace and isolate every single case that arises, so as to halt the spread of the virus [4].

The USA (US) has extended travel restrictions from certain COVID-19 affected countries like Brazil, UK (UK) and China [5]. Pandemic fatigue is being experienced in many countries [6,7]. Economic crises, stress and fear are threatening to become the next pandemics [6,7]. The tension is palpable and many are getting agitated as anti-lockdown protests spread across Europe [8,9].

A growing number of scientists are increasingly talking of the need to learn to live with this virus [10–12]. What that means in practical terms, is not very clear. Some of the reasons for enforcing lockdowns have been to curb the spread of the virus and prevent a health system overload. Many countries that have health systems previously thought to be world class have also been tested, pushed to the edge and in some ways found wanting [13,14]. The pandemic took all countries by some surprise. China, Italy, Spain, France, Brazil, Mexico, USA, and the UK were hit hard. South Africa and recently Zimbabwe, have all borne the brunt of the pandemic, particularly the second wave from around Dec 2020 to Jan 2021. Currently (March 2021), the pandemic is plaguing both the northern and southern hemispheres, with new variants emerging in Brazil, UK, South Africa and India. Many people are working mostly from home and most universities and some schools have moved to online learning.

Societal issues, old versus young, value of life vs economy and science vs social media

The pandemic has made existing societal chasms more visible [15,16]. Unfortunately, too quickly and too often, dichotomies are created and amplified by the fact that infections and diseases are unevenly distributed across age groups, space and time. The divide between the young and the old was reported at the beginning of the pandemic[15]. Young people initially thought they were less vulnerable and hence saw lockdowns as measures to prevent the vulnerable old, from getting the disease. Other older people saw the restless young as the ones refusing to adhere to social distancing rules, thereby spreading the disease [15]. Our interconnectedness and the value of life iitself have been tested by the pandemic. Debates on saving lives versus economic interests have not been exhaustive either[17]. The need for information, on a novel virus SARS-CoV-2, led to a scramble to know what the latest facts are, what works best and where, pitting science (studies take time to generate knowledge) against social media. As is the nature of scientific inquiry, the multiple views of scientists on the same issues, complicates the matter even further[17]. The dichotomy carries on with pro-vaccine and anti-vaccine groups to date.

### Living with the virus and emergent variants

The proposed strategies range from aiming for No-Covid [18], to living with the virus. Others argue that the virus is new, not like any other we have fought before, calling for novel ways of containing the virus. On the other hand, calls for living with the virus are becoming more prominent, based on lessons from previous pandemics [10–12,19]. Irrespective of standpoint, pandemic fatigue is setting in while new variants are being discovered. It`s urgent and unprecedented. What does living with the virus mean in real terms? Which measures need to be put in place, maintained and what role does vaccination and the overall concept of herd immunity play? COVID-19 is not that simple, since it is both an infection with a severe acute respiratory syndrome and an array of non-communicable diseases too [20]. Contexts differ and countries across the globe will respond differently. The purpose of this viewpoint is to stimulate debate on how pandemic proof health care systems look like.

### Health system and health care system definitions

A health system is part of the overall social system in any setting and is defined as all organizations, institutions and resources that produce actions whose primary purpose is to improve health [21]. A health care system on the other hhand is defined as institutions, people and resources involved in delivering health care to individuals [21]. For clarity reasons, we are going to use the term health care system (institutions, people, and resources) throughout this paper. See Table 1

**Table 1.** Summary of policy, structural and operational issues that might need attention

	Institutions	Resources	People
Governance level High- and low-income countries	Revised law of epidemics Cooperation between low- and high-income countries Laws, regulations and guidelines on preparedness Revisiting the role of private sector providers Establishment of national and internationally linked surveillance-response systems	Investment in resources, infrastructure and telemedicine Investment in surveillance systems including genetic sequencing	Laws to ensure health systems have HR development plans to assure the constant supply of health care workers Valued health care workers with more pay and better working conditions
Policies and Strategy level High- and low-income countries	New policies and strategies to take services to where the people are- homes Renewed approaches for health promotion and prevention in societies and populations	Policies that promote local production of resources e.g. PPE that is environmentally friendly Development of environmentally friendly products particularly disinfectants	Review policies of health cadres- cadre for epidemic preparedness and surveillance- response Provision of satellite testing and vaccination centres
Service delivery/operational level High- and low-income countries	Expansion of telemedicine from triage, diagnosis and follow up services covering all conditions (acute to chronic)? taking care where the people are- through home-based care services? Acknowledgement and reinforcement of health as wealth Health promotion and prevention services provided to people in their homes Expansion of services to include preventive and promotive mental health services? In what format? Online, face to face formats or home visits? Establishment of research units- Ongoing institutional studies to ascertain treatment guidelines and use of off-label products for unknown disease entities in observational clinical studies **Science vs Social media** -will institutions adapt and become centres of knowledge dissemination- telemedicine and all possibilities of e- and m-health and provide knowledge to people at home, on what symptoms to look out for, where to test, how to self-care etc? for other conditions too and not only COVID-19	Functional supply chain systems Testing kits Vaccines for all age-groups	Develop more Online training and reference resources for staff. Home based vaccination programmes Mental health support for staff to reduce stress, burn out, debriefing and counselling services
Infrastructure High- and low-income countries	Structural changes e.g. air filters, ventilation systems, patient flows. Adaptation of waiting rooms to have windows that can be opened Disinfection and aeration of rooms	Storage facilities to stores sufficient material and prepare for future pandemics	Mobile vans etc to take care into the homes, dialysis teams etc
Staff and training High- and low-income countries	Systems thinking and within systems understand essence and processes of surveillance-response	Systems thinking and within systems understand essence and processes of surveillance-response	New level cadre, training and within systems understand essence and processes of surveillance-response Systems thinking

## What does a pandemic proof health care system look like?

### Institutions

The institutions include health facilities; among others clinics, medical practices and hospitals. The pandemic challenges us not only on the conceptual levels but very practically as discussed below.

### Curative services

What kind of health care services will institutions provide? Are we going to see a need for the expansion of telemedicine from triage, diagnosis and follow up services covering all conditions (acute to chronic)? Are we going to see institutions moving into taking care where the people are- through home-based care services? Will institutions adapt and become centres of knowledge dissemination- telemedicine and all possibilities of e- and m-health and provide knowledge to people at home, on what symptoms to look out for, where to test, how to self-care etc? for other conditions too and not only COVID-19? What telemedicine services will be incorporated into every day practice after the pandemic? Telemedicine expansion seems inevitable-are we going to see big health system investments into telemedicine technology in both low-and high-income countries? See Table 1 above

### Preventive and promotive health services

Staying at home has brought about new opportunities and challenges too. Lockdowns have also meant closure of sport facilities including gyms for those that afforded these. Fitness, diet and exercise choices have been left mostly to individuals. The pandemic has demonstrated that having an underlying condition like diabetes, hypertension or obesity predisposes one to complications, long hospital stays and mortality. Due to the above reasons, COVID-19 has been termed a syndemic rather than a pandemic [20]. Addressing COVID-19 is indeed complex. This entails addressing obesity, diabetes, cancer, chronic respiratory diseases and hypertension [20], notwithstanding the broader socio-economic challenges.

Health is wealth [22] – are we going to see a more concrete recognition of this notion? Are institutions going to tap into this and provide preventive and promotive health services like boosting one’s immune system through diet, exercise and supplements, in different formats, online, face to face or through home visits? See Table 1 above

### Mental health services

Mental health issues during the pandemic are threatening to become the next pandemic. Stress and mental health issues related to lockdowns affect everyone but tend to affect the weaker segments of the society more severely. The poor often have limited space to accomodate homeoffice and home schooling, not to mention the additional weight of economic effects and job insecurities [23,24] .

### Long COVID

Some people that recovered from COVID-19 are still experiencing its effects-long covid with some now in need of dialysis and others developing diabetes. The uncertainties and anxiety accompanied by confinement to homes is creating new health needs [20,25].

Our societies need hope [20]. Drugs or vaccines alone cannot solve the syndemics we are faced with. Are we going to see athe systems thinking approach that incorporates housing, health, employment, food, environment and educational issues [20]?

Are institutions going to expand their services to include preventive and promotive mental health services as well as mobile dialysis teams? In what format? Online, face to face, home visits or blended? See Table 1 above.

### Infrastructure

Health care system infrastructure is often built with centralized ventilation systems that could promote the spread of viruses such as COVID-19. Structural change e.g. air filters and a re-think on patient flows seem imminent. The waiting rooms might need to be adapted to having preferably few persons at a time and the rooms be disinfected and aerated after each patient. Some waiting rooms have no windows that can be opened-what will happen to these? In some countries in the North, winters are hard and heating is expensive. Can practices and institutions afford to open windows after each patient in winter? If high volumes of people fall ill at the same time, institutions might run out of the beds and even oxygen as was experienced by some countries. So, do the pandemic proof institutions have to be restructured, to take health care to where the sick are-to homes rather? See Table 1 above

## Resources

### Disinfectants and PPE

Disinfectant use is one component among strategies being used to curb the spread of the virus. Are we going to see development of environmentally friendly disinfectants or is the world going to wake up to other multi-resistant bugs due to indiscriminate use of disinfectants? Many institutions had COVID-19 wards or infectious disease units as in some hospitals in Africa [26]. If COVID-19 were to be everywhere-does that mean all health care workers in all units will need to wear full protective personal equipment (PPE) gear daily? Is that affordable? The world is already drowning in plastic. Our seas are flooded. Can we afford to keep producing daily tonnes of plastic through disposable PPE now and in future pandemic states? Are we going to see more local production of sustainable and reusable PPE? See Table 1 above

### Testing kits

There are initiatives and moves towards self-testing. That might become the order of the day. Who should test, how often and when, needs to be answered? The PCR test costs to date have been borne by the governments in some countries. Will that remain so? This is important, as the cost of tests can hinder testing, bearing in mind the economic strains borne by many, that have suffered in multiple ways during the pandemic. See Table 1 above

### Vaccines

With whole populations at risk, are vaccinations going to become the norm? Are we going to see institutions adapting to include ongoing vaccination services across all age groups, from the infants to the elderly? Viral mutations occur and they do threaten the efficacy of vaccines. Are we going to see investment into surveillance systems including genetic sequencing and parallel investments in second and third generation vaccines? See Table 1 above

## People

A system is made up of people and a system is as good as the people in the system. The world is already experiencing a health workforce crisis. Health care workers resigned due to fear of getting infected at the height of the pandemic. PPE shortages were to blame in some instances and this was blamed on pandemic unpreparedness, as very few if any, anticipated its extent and magnitude. Fatigue and burn-out were experienced by many and some intensive care unit (ICU) nurses ended up looking after 5 patients as compared to a 1:1 ratio. Some nurses were even assigned to work in unfamiliar units like intensive care unit (ICU) without any training [27]. The impact this had on quality of care was attributed to high mortality rates in some cases [28,29]. In the context of such health worker shortages, are we soon going to see robots caring for people at home and in institutions-robotic telemedicine? See Table 1 above

**Figure 1.**
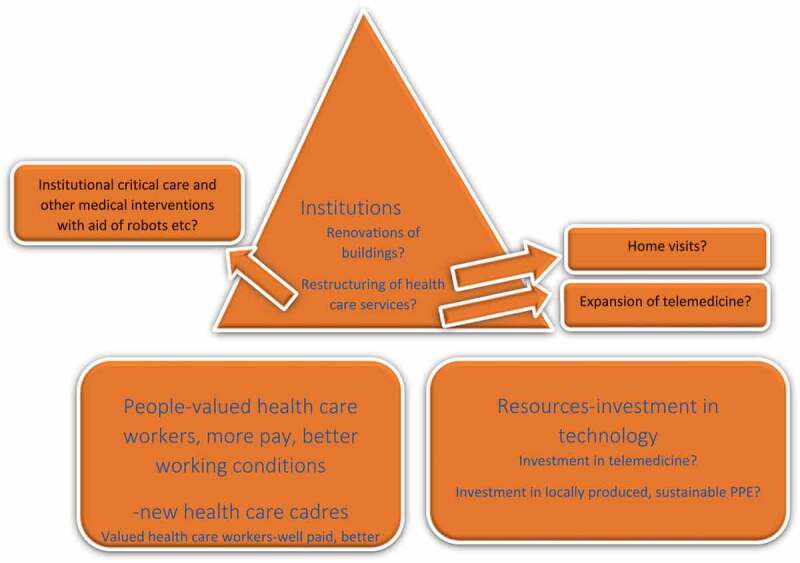
A pandemic proof health care system

What needs to happen to make our health care systems pandemic proof wwith regard to health care workers? How can the constant supply of health care workers be ensured? Online training of low level cadres, like the health care workers and clinical associates in Africa [30]? Are we going to see more health care workers trained to provide home based care and home visits and teach patients self-care, not only in COVID-19 contexts but other conditions as well? [30]. Many countries applauded the health care workers during the peak of the first wave. Are we going to see health care workers valued more, with better pay and working conditions? See figure 1 for the depiction of a pandemic proof health care system.

## Both low- and high-income countries were affected

The poor always pay more but the divide between the low and the high-income countries was blurred by the virus. Both hemispheres were ravaged, with some high-income countries suffering more casualties as compared to the low-income countries to date. The vaccine availability and roll-out might change the dynamic particularly, the COVAX facility. Are we going to see more cooperation between the low- and high-income countries in future? See Table 1 for a summary of policy, structural and operational issues.

## Conclusion

The purpose of this view point is to stimulate debate. SARS-CoV-2 has changed our world in multiple ways. We wake up each day to a new normal. What does a pandemic proof health care system look like, is meant to stimulate debate and create an impetus for changes that our health care systems might need to make, as the pandemic drags on and new variants emerge. Assessing the course of the pandemic also shows us that the changes to be made in and for the health systems need to be tailored to the respective settings; no ‘one size fits all’ approaches are possible. High-, middle- and low-income countries will respond differently. The health system implications as outlined are far reaching, ranging from conceptual, policy, considerations of effective surveillance-response systems to the very operational and structural issues including health infrastructure construction in relation to patients’ needs. The pandemic has clearly revealed policy, structural and functional gaps; a chance and an opportunity. Besides all this, the health care system is part of the larger socio-ecological system in any country, and hence the overall challenge remains in identifying environmentally sustainable ways towards more effective and equitable health systems, sensitive to individual as well as societal needs. The pandemic is here and more pandemics are expected in future. Irrespective of aiming for No Covid, Zero Covid or for living with the virus, or being a high-, middle- or low-income country, what needs to be done as both uncertainty and the pandemic linger on? We acknowledge that this is indeed a mammoth task. What does a pandemic proof health care system look like, is meant to stimulate debate.

## Paper context

Pandemic fatigue is setting in. The tension is palpable and many are agitated as anti-lockdown protests spread across Europe. Scientists are divided. Irrespective of aiming for No Covid, Zero Covid or for living with the virus, being a high-, middle- or low-income country, what needs to be done as both uncertainty and the pandemic linger on? The purpose of this manuscript is to stimulate debate on what a pandemic proof health care system looks like.
